# Towards Advances in Molecular Understanding of Boric Acid Biocatalyzed Ring-Opening (Co)Polymerization of δ-Valerolactone in the Presence of Ethylene Glycol as an Initiator

**DOI:** 10.3390/molecules26164859

**Published:** 2021-08-11

**Authors:** Khadar Duale, Piotr Latos, Anna Chrobok, Adrian Domiński, Magdalena Martinka Maksymiak, Grażyna Adamus, Marek Kowalczuk

**Affiliations:** 1 Centre of Polymer and Carbon Materials, Polish Academy of Sciences, 34, M. Curie-Sklodowska St., 41-819 Zabrze, Poland; adominski@cmpw-pan.edu.pl (A.D.); mmaksymiak@cmpw-pan.edu.pl (M.M.M.); gadamus@cmpw-pan.edu.pl (G.A.); 2 Department of Chemical Organic Technology and Petrochemistry, Silesian University of Technology, Krzywoustego 4, 44-100 Gliwice, Poland; piotr.latos@polsl.pl (P.L.); Anna.Chrobok@polsl.pl (A.C.)

**Keywords:** poly (δ-valerolactone), boric acid, oxetane-2-ones (β-lactones), metal free catalyst, 6-methyl-6-ε-caprolactone, 7-methyl-2-Oxepanone, polymer MS-MS studies, biodegradable polymers, biocatalyst, baeyer-villiger oxidation

## Abstract

Following our previous studies on the molecular level structure of (co)oligoesters obtained via anionic homo- and co-polymerization of novel β-substituted β-lactones, prepared by the atmospheric pressure carbonylation reaction of respective epoxides, the boric acid biocatalyzed ring-opening (co)polymerization of δ-valerolactone has been studied. As a co-monomer the 6-methy-ε-caprolactone, prepared by the one-pot oxidation of respective alcohol, and ethylene glycol as polymerization initiator were used. The obtained copolymers were characterized by ^1^H-NMR, GPC and ESI-MS, respectively in order to confirm their chemical structures and identity. Subsequently, tandem mass spectrometry (MS-MS studies) via collision-induced dissociation were utilized to characterize the fragmentation pattern. ESI-MS and NMR analyses confirmed the formation of random linear copolymer chains composed of different polyester repeat units. MS-MS experiments showed that fragmentation proceeds via ester bound cleavage along the (co)polyester chains. The innovative aspect of this contribution is related to the elaboration of the telechelic (co)polymers end-capped with hydroxyl end groups and well-defined molecular architectures, which could facilitate the development of new flexible macromolecular systems for potential biomedical applications.

## 1. Introduction

Coherent activities in organic chemistry and polymer sciences reflect the development of new polymeric materials for specific applications. In collaboration with Professor Janusz Jurczak, a procedure for the epoxide carbonylation reaction and the possibility of large-scale production of optically active oxetane-2-ones were explored [1]. Oxetane-2-ones (β-lactones) are attractive intermediates in organic synthesis, not only because they are often presented as a structural unit of biologically active compounds, but they also find a myriad of applications in biopolymer synthesis [2]. Likewise, further collaborative studies on bioactive anisic acid-conjugated oligomers, in which the bioactive molecules were covalently bonded as pendant groups along an oligomeric backbone, demonstrated the ability to synthesize homo- and (co)oligoesters with a bioactive moiety by anionic ring-opening polymerization (AROP) of β-substitutes lactones [3]. In addition, the molecular structure of these bioactive (co)oligoesters was also demonstrated, as well as their ability in the potential application in cosmetology [4,5,6]. Such structural characterization requires sophisticated analytical techniques utilizing NMR, GPC, DSC, TGA, and MS. However, due to its superior resolution, MS has become an important analytical tool routinely used for various types of organic, biological and polymer analysis [7,8,9,10,11]. In particular, tandem mass spectrometry, which uses the fragmentation of mass-selected parent ions, has been utilized efficiently to determine macromolecular connectivities and sequences analysis such as branches, crosslinks, distinctions between isobaric and isomeric species, chain connectivity, isomeric purity, and end group compositions analysis of (co)polyesters, of both natural and synthetic origin [11,12,13,14,15]. End groups play an important role in understanding the mechanism of β-lactone ring-opening polymerization and the subsequent chain propagation reaction so that tailored polymers with the desired architecture and molar mass can be designed [16].

For ring expanded monomers, boric acid catalyzed homopolymerisation of δ-valerolactone and copolymerisation of δ-valerolactone with ε-caprolactone initiated by benzyl-alcohol have previously been reported by us and Ren et al., respectively [17,18]. The most widely used industrial catalysts for ring-opening polymerization of cyclic esters is tin (II) octanoate [Sn (Oct)_2_]; however, several types of other metal catalysts such as aluminum alkoxides, calcium ammoniate, phosphasalen indium and zinc butoxides are also used [19,20,21,22,23,24]. Consequently, the use of non-metallic boric acid organocatalyst has not been exploited extensively in the ring expansion reaction of higher lactones polymers; however, it alleviates problems with the rigorous work-up required to obtain a polymer sample free of a residual trace of metals, which could interfere in a bioassay [25]. Boric acid, due to its environmentally benign physicochemical properties combined with its easy commercial availability, has recently gained exceptional interest in organic synthesis as a substitute for conventional acid catalytic materials. In this context, boric acid has proved to be particularly versatile, having been shown to catalyze an extensive array of organic transformations, such as halogenation reaction, amidation, esterification, the metal-organic framework, Aza-Michael addition, bromination of a variety of organic substrates, ipso-hydroxylation, and transesterification [26,27,28,29,30,31,32].

The present study focused on hydroxy-telechelic (co)oligoesters prepared via boric acid biocatalyzed ring-opening (co)polymerization of δ-valerolactone (dVL). Whereas ethylene glycol has been applied as a polymerization initiator and 6-methyl-ε-caprolactone (mCL) as a comonomer. This later monomer was recently prepared by some of us through the one-pot Baeyer–Villiger oxidation of respective cyclic ketone. The standard procedure uses peroxyacids as the oxidizing agent, which are highly effective and selective [33]. The obtained telechelic (co)polymers were characterized by ^1^H-NMR, GPC, and ESI-MS, respectively, in order to confirm their chemical structures and composition. Consequently, tandem mass spectrometry was used to characterize the fragmentation pattern. Thus, structural studies at the molecular level confirmed the formation of random linear copolymer chains consisting of different repeating units, while the presence of hydroxyl groups at both (co)polymer ends was identified by MS-MS experiments.

## 2. Results and Discussion

**Scheme 1:**
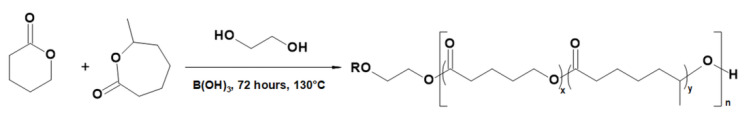
The ring-opening polymerization of δ-valerolactone and 6-methyl-ε-caprolactone initiated by ethylene glycol with the presence of boric acid as a catalyst to activate the monomer, R = H represents macromolecules with ethylene glycol incorporated as an end groups; R represents the second arm of PdVL homopolymer or the copolymer P (dVL-co-mCL) with ethylene glycol incorporated as a bridge to the (co)polymers chains.

The objective of the research described in this paper was the synthesis and in-depth characterization of poly (δ-valerolactone) (PdVL) homopolymer and random poly (δ-valerolactone-co-6-methyl-ε-caprolactone) P(dVL-co-mCL) copolymers at molecular level structure studies. The respective homo- and copolymers were synthesized via boric acid biocatalyzed ring-opening polymerization of δ-valerolactone and its copolymerization with 6-methyl-ε-caprolactone, with the aid of ethylene glycol as a polymerization initiator. The obtained random oligomers contain hydroxyl groups at both chain ends of poly (δ-valerolactone) homopolymer and poly (δ-valerolactone-co-6-methyl-ε-caprolactone) (co)polymer, respectively. Moreover, based on the polymerization mechanism, it could be expected to obtain homo- and copolymers with various chemical structures of the chains: (i) homo- and copolymer macromolecules containing ethylene glycol incorporated as a “bridge” into the oligomer chain and (ii) homo- and copolymer macromolecules with ethylene glycol introduced as an end group (Scheme 1). The obtained polymer samples were preliminarily characterized by gel permeation chromatography (GPC), proton nuclear magnetic resonance ^1^H-NMR, and particularly by ESI tandem mass spectrometry (ESI-MS/MS) techniques. The influence of the additional CH-CH_3_ group in the linear polymer chain that mCL oligomers present compared to dVL oligomers is of interest in terms of fragmentation pathways. This aspect was one of the objectives of the present study and required further investigation.

The results of characterization of the polymers obtained by GPC are summarized in Table 1.

### 2.1. NMR

**Figure 1:**
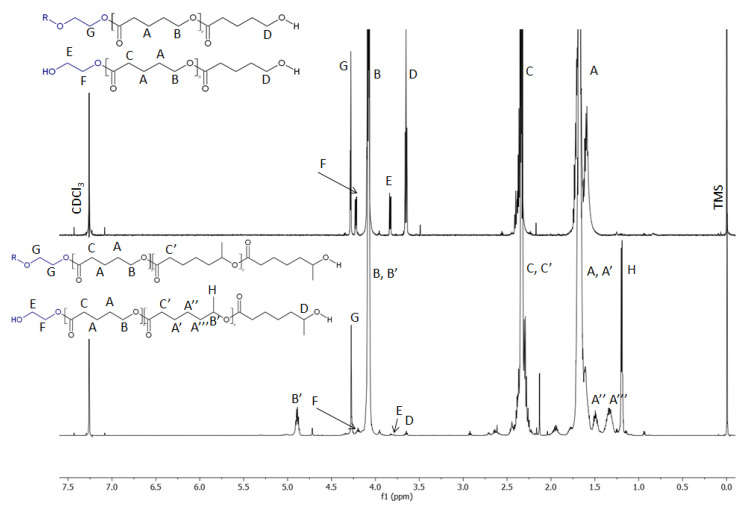
^1^H NMR spectrum (CDCl_3_, 600 MHz) of: (1) PdVL (top), and (2) P(dVL-co-mCL) (bottom); R represents the second arm of the copolymer P(dVL-co-mCL).

NMR measurements were carried out to provide detailed information about the chemical structure of the obtained polyesters. The chemical structure of obtained polyesters in this study was differentiated by ^1^H NMR and presented in Figure 1. In ^1^H NMR spectrum of the obtained poly (δ-valerolactone) (Figure 1, top) the signals at δ = 1.65, 2.34, and 4.08 ppm were attributed as respective protons of dVL repeating unit. Signal at δ = 3.65 ppm was assigned to the protons of CH_2_OH end group. The two signals at δ = 3.83 and 4.21 ppm correspond to the ethylene glycol units at the end group, while the signal at δ = 4.28 ppm is showing the presence of ethylene glycol incorporated as a bridge between two dVL homopolymer chains. The structure of P(dVL-co-mCL) copolymer was also verified by ^1^H NMR as shown in Figure 1 (bottom). All of the main characteristic signals of (δ-valerolactone) units and 6-methyl-ε-caprolactone units (δ = 1.2, 1.33, 1.49, 4.89 ppm) are clearly detected. The above results confirm the successful ring-opening copolymerization of dVL and mCL initiated by EG, using boric acid as catalyst.

### 2.2. ESI-MS

Analyses by ESI-MS were conducted on homopolymer of poly (δ-valerolactone) and random (co)polymers of (δ-valerolactone-co-6-methyl-ε-caprolactone) and containing hydroxy groups at both polymer chain ends (Sample 1-PdVL and 1-P(dVL-*co*-mCL), Table 1), obtained after the ROP (co)polymerization. The results for each (co)polymer type are discussed separately below.

#### 2.2.1. Structural Studies of the Homo-Oligoester PdVL by ESI-MS/MS

**Figure 2:**
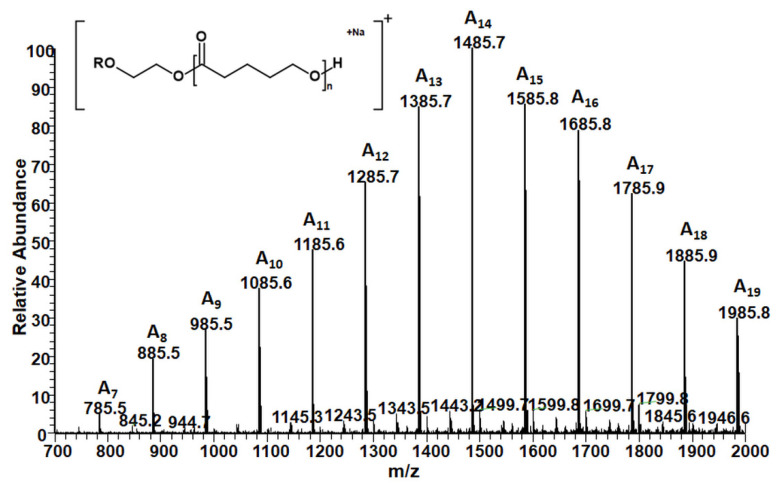
ESIMS spectrum (positive-ion mode) of PdVL macromolecules. R = H represents macromolecules with ethylene glycol incorporated as an end groups; R represents the second arm of PdVL homopolymer incorporated to ethylene glycol as a bridge of the polymer chains.

The ESI mass spectrum of PdVL oligomers obtained here is depicted in Figure 2. One main and most abundant set of ions with a peak-to-peak mass increment of 100 Da was observed in the mass spectrum. The structures of the end groups and repeating units can be inferred, based on the mass assignment of singly charged ions observed in the mass spectrum. Analysis by ESI-MS was conducted on hydroxy-telechelic poly (δ-valerolactone) containing primary hydroxy groups at both polymer chain ends (Sample 1-PdVL, Table 1), obtained after the ROP reaction. The positive ESI-MS spectrum (Figure 2) indicates one main set of macromolecules of singly charged ions which corresponds to sodium adducts of dVL oligomers present in the sample.

Based on the polymerization mechanism, the series A can correspond to two kinds of sodium adducts of PdVL oligomers terminated by hydroxyl end groups and containing ethylene glycol incorporated as a “bridge” into the oligomer chain or as a terminal group. Despite the different structure of the chain of these two types of oligomers, their average molar masses are the same, therefore the spectrum shows one isobaric series of singly charged ions (A).

**Figure 3:**
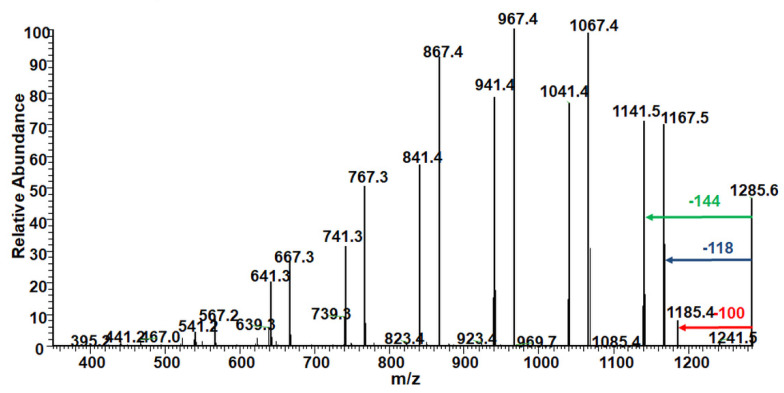
ESI-MS/MS spectrum of the sodium adducts of poly (δ-valerolactone) macromolecules at *m*/*z* 1285.

**Scheme 2:**
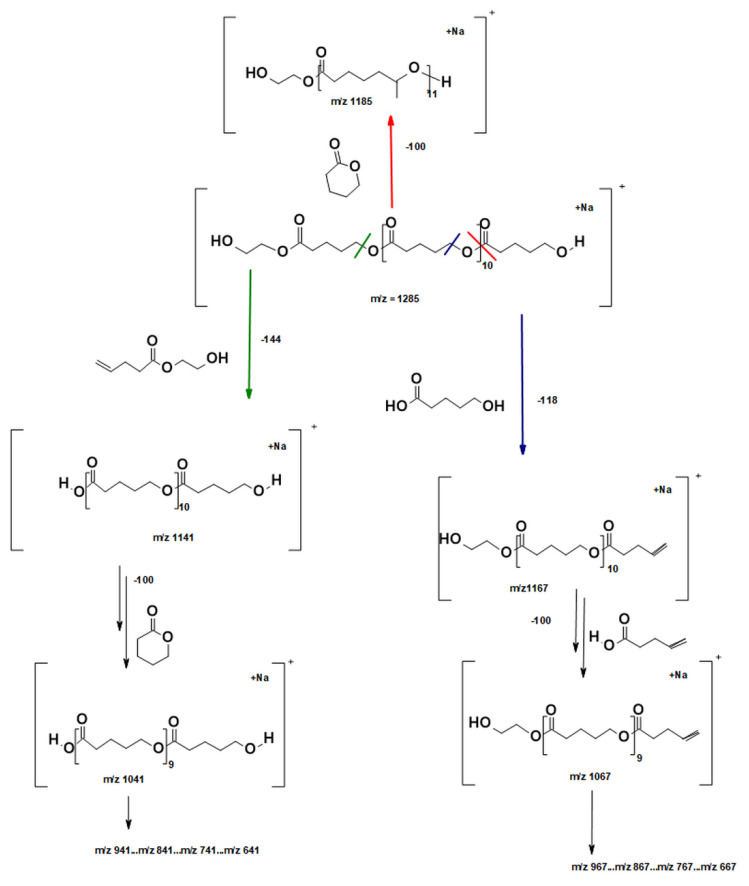
Possible fragmentation pathways for sodium adducts of telechelic PdVL oligomers with ethylene glycol incorporated as an end group of polymer chain at *m*/*z* 1285.

**Scheme 3:**
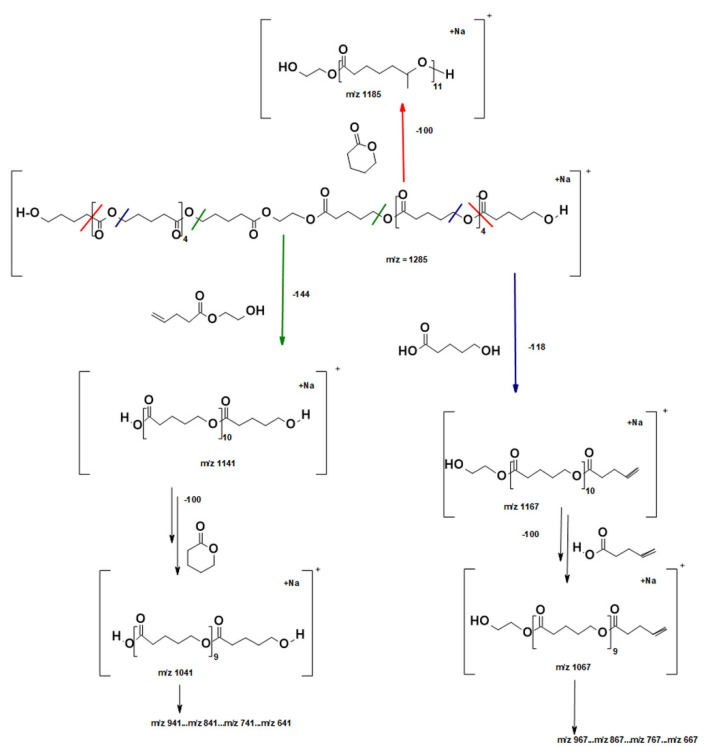
Possible fragmentation pathways for sodium adducts of and hydroxy-terminated PdVL oligomers with ethylene glycol incorporated as a “bridge” inside the polymer chain at *m*/*z* 1285.

Figure 3 shows the ESI-MS/MS spectrum of the ion at *m/z* 1285 belonging to the main and most abundant series A. The theoretical fragmentation pathways of the two kinds of ions belonging to series A, and the PdVL telechelic oligomers and containing ethylene glycol incorporated as an end group or as a “bridge” in the homopolymer chains are shown in Scheme 2 and Scheme 3, respectively. The fragmentation of these oligomer parent ions can occur as a result of random breaking of the ester bonds along the oligomer chain, but also as losses of neutral molecule of monomer dVL [17,34,35,36,37]. A comparison of the fragmentation spectra and the theoretical paths of the ion fragmentation shows that all theoretically predicted structures of the product ions are shown at the spectrum, which confirms the structure of the obtained PdVL macromolecules (Figure 3 and Scheme 2 and Scheme 3).

#### 2.2.2. Molecular Level Studies of the Oligoester P(dVL-co-mCL) Performed by ESI-MS^n^

**Figure 4:**
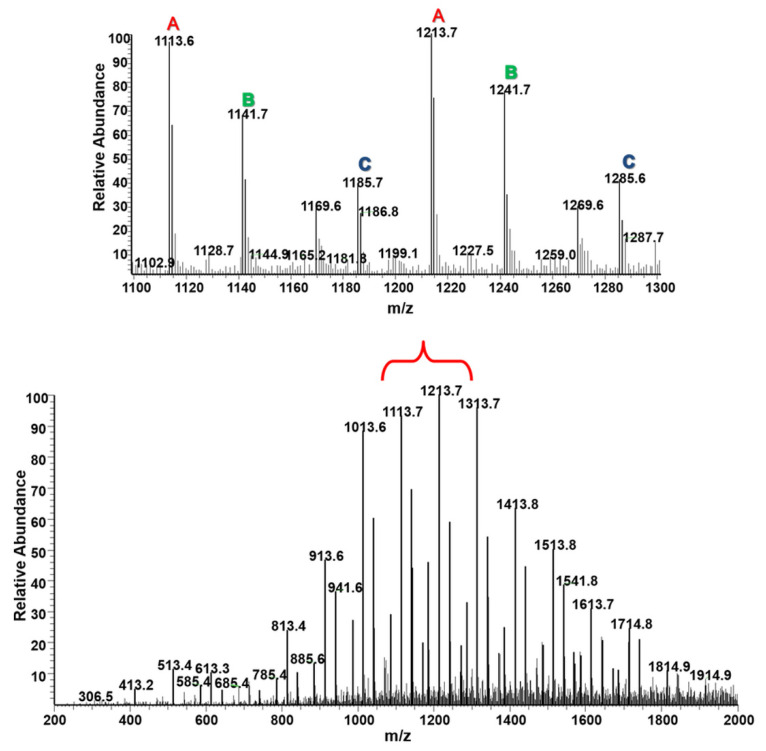
ESI-MS spectrum of the sodium adducts of poly (δ-valerolactone-valerolactone -co-6-methyl-ε-caprolactone) macromolecules.

Analysis by ESI-MS was conducted on poly (δ-valerolactone-6-methyl-ε-caprolactone) (Sample 1 P(dVL-co-mCL), Table 1). The ESI mass spectrum of P(dVL-*co*-mCL) (co)oligoester obtained here together with spectral expansion in the mass range *m*/*z* 1100–1300, are depicted in Figure 4. The structures of the end groups and repeating units can be inferred based on the mass assignment of singly charged ions observed in the mass spectrum. The positive ESI-MS spectrum (Figure 4) indicates the presence of three series of singly charged ions A, B, and C which correspond to three main types of oligomers present in the copolymer sample. The observed ions can be assigned structurally based on their *m/z* value. The most abundant sodium adduct (belonging to series A) in the ESI-MS spectrum, and appears in the mass spectrum at *m*/*z* 1113 was assigned to the P(dVL-*co*-mCL) copolymer with one mCL unit and terminated by hydroxyl end groups at both ends. The molar mass of the sodium adducts of (δ-valerolactone) oligomer terminated by hydroxyl end groups can be theoretically calculated by the following equation *m*/*z* = (n × 100) + 128 + 61 + 1 + 23. (Where: 128 is the molar mass of mCL, 61 is the molar mass of the end group derived from ethylene glycol, while 100 corresponds to molar mass of δ-valerolactone unit and n is the degree of polymerization, 1 is the mass of proton and 23 is the mass of sodium).

**Scheme 4:**
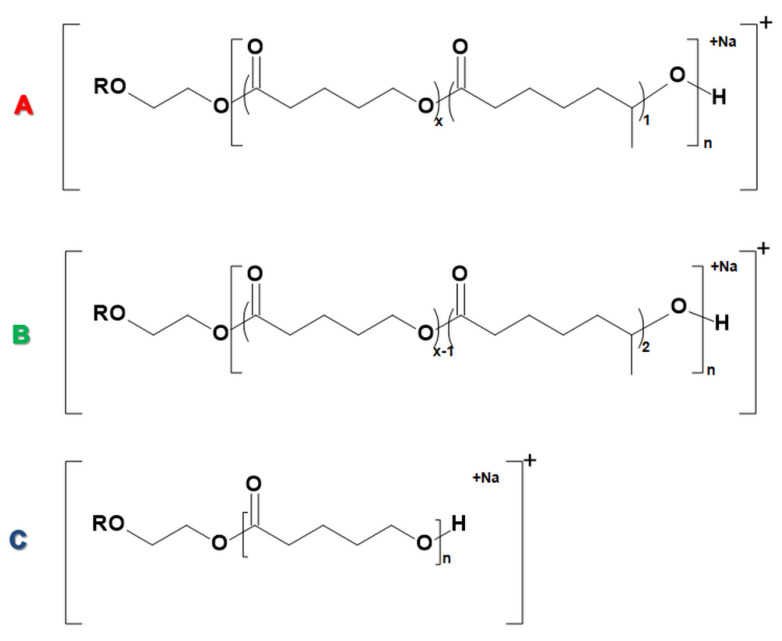
The structures of the ions observed in ESI-MS of poly (δ-valerolactone-6-methyl-ε-caprolactone) macromolecules. R = H represents macromolecules with ethylene glycol incorporated as an end groups; R represents the second arm of PdVL homopolymer or the copolymer P(dVL-co-mCL) and/or represents macromolecules containing ethylene glycol incorporated as a bridge to the (co)polymers chains.

**Figure 5:**
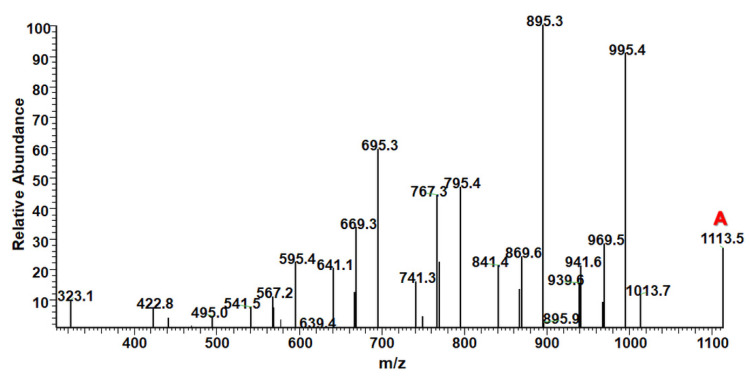
ESI-MS/MS spectrum of the sodium adducts of poly (δ-valerolactone–6-ε-methyl-caprolactone) macromolecules and containing one mCL comonomer unit at *m*/*z* 1113.

The sodium adduct (Series B) located at *m/z* 1141 in the spectrum can be assigned to P(dVL-co-mCL) copolymer with two mCL units incorporated into the polymer chain and this oligomer can be calculated by: *m/z* = (n × 100) + 61 + 2 × 128 + 1 + 23. Finally, the sodium adduct (series C) corresponding to the oligomers at *m*/*z* 1185 represent PdVL homopolymer terminated by hydroxyl groups at both ends and appear in the spectrum at *m/z* = (n × 100) + 61 + 1 + 23. The ESI-MS^2^ fragmentation analyses of selected ions are presented in Figure 5. The structures of the ions observed in ESI-MS of poly (δ-valerolactone–6-methyl-ε-caprolactone) copolymers are depicted in Scheme 4. Similarly to in the case of PdVL homopolymers observed in the mass spectrum, each of the two series of ions A and B represent two kinds of P(dVL-*co*-mCL) copolymer macromolecules terminated by hydroxyl end groups and containing ethylene glycol incorporated as a “bridge” into the oligomer chain or as a terminal group. Additionally, series C represents PdVL two types of homopolymer macromolecules.

#### 2.2.3. Fragmentation Studies of P(dVL-co-mCL) Copolymer with One mCL Comonomer Unit

**Scheme 5:**
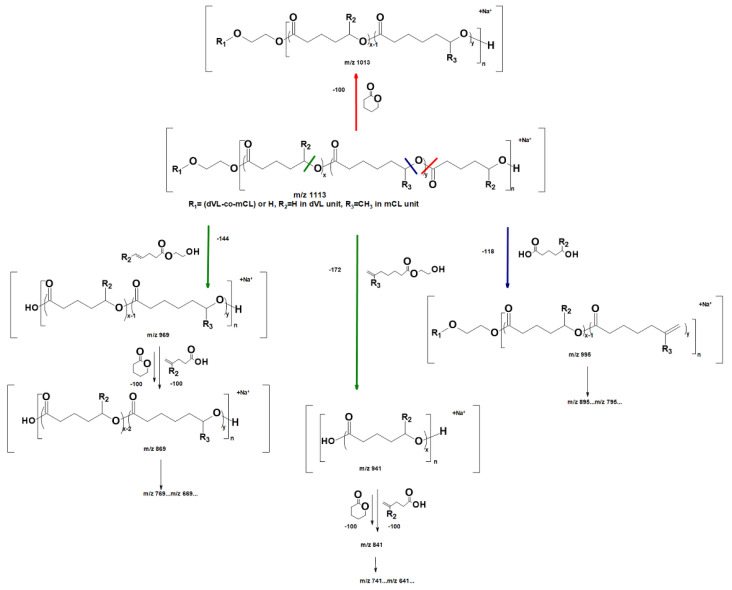
Possible fragmentation pathways for sodium adducts of poly (δ-valerolactone–6-methyl-ε-caprolactone) macromolecules at *m*/*z* 1113; R_1_ = P(dVL-*co*-mCL) or H, R_2_ = H in dVL unit, R_3_ = CH_3_ in mCL unit.

Figure 5 illustrates the ESI-MS/MS spectrum of the sodium adducts of hydroxyl- terminated poly (δ-valerolactone–co–6-methyl-ε-caprolactone) oligoesters at *m*/*z* 1113 and belonging to the series A. The fragmentation of the parent ion at *m*/*z* 1113 highlights the formation of four sets of fragment pathways as the ESI-MS/MS experiments indicate that the oligomer is fragmenting from both ends. The first fragmentations series represents the attack of hydroxyl end groups on adjacent carbonyl carbon and subsequent loss of neutral molecule of cyclic 5-valerolactone (100 Da) (see Scheme 5). This is a form of back biting reaction formed by the release of neutral molecule of cyclic *δ*-valerolactone of 4-pentenoic acid and has also been observed in our previous studies on benzyl alcohol terminated dVL [17]. It appears that the fragmentation stops at that point as no further loss from the *m*/*z* 1013 fragment ion is observed in this series. The fragment ion at *m*/*z* 995 is the next series and is formed due to the loss of 5-hydroxyvaleric acid (118 Da). Additional loss of 118 Da is not observed for this series, however loss of 100 Da corresponding to neutral molecules of *δ*-valerolactone or 4-pentenoic acid is seen at *m*/*z* 895, 795, 695, 595 and 495. The respective macromolecule also shows random breakage of ester bonds along the polyester chain, and in this case forms 2-hydroxyethylpentene-4-enoate (144 Da) and the loss of 172 correspond to 2-hydroxyethyl 5-methylhex-5-enoate (172 Da). Similarly, further fragments of neutral ions are seen at *m*/*z* 841, 741, 641, 541 and 441 in this series. Scheme 5 presents the possible fragmentation pathways for the sodium adduct of series A and represented by an ion at *m/z* 1113. The proposed mechanism is based on our knowledge of the fragmentation pathways of different types of polyester chains [34,35,36].

The results of the fragmentation studies of P(dVL-co-mCL) copolymer with two mCL comonomer units clearly demonstrate that the mCL units are incorporated along with the co-polymer chains. It is worth pointing out that the fragmentation of P(dVL-co-mCL) copolymer with two mCL comonomer units takes place according to the scheme presented for P(dVL-co-mCL) copolymer with one mCL comonomer unit with even more complex pathway.

## 3. Conclusions

The result discussed above deliver new knowledge regarding the ring expanded monomers (co)polymerization and fragmentation pathways. Well-defined dVL homopolymer and copolymers were directly synthesized via ROP in the presence of ethylene glycol and boric acid as organocatalyst, respectively. Subsequently, the molecular weight, architecture, and fragmentation pathway of obtained macromolecules were characterized using GPC, NMR, and ESI-MS/MS analyses. The coherent NMR and tandem mass spectrometry evaluation of the obtained (co)polymers structure at the molecular level was provided. The results of the fragmentation studies of P(dVL-co-mCL) copolymer with one and two mCL comonomer units clearly demonstrate that the mCL units are incorporated along with the copolymer chains. The evidence of the versatility of ESI-MS/MS technique demonstrates the convenience of this method for the evaluation of (co)polymer fragmentation pathways and end-group analysis. The further extension of boric acid use as a safe and effective organocatalyst in lactones (co)polymerization has been given.

## 4. Materials and Methods

δ-Valerolactone (δ-VL 99% purity), purchased from TCI, (Brussels, Belgium) was freshly distilled from CaH_2_. Ethylene Glycol (99% purity), from Alfa Aesar, (Thermo Fisher, Karlsruhe, Germany) and boric acid (B (OH)_3_, (99.5% purity) purchased from Sigma-Aldrich, (Merck, Darmstadt, Germany) were used as received. 2-methylcyclohexanone (99% purity), purchased from Sigma-Aldrich, (Merck, Darmstadt, Germany), 3-Chloroperbenzoic acid (*m*-CPBA, ≥70% purity), purchased from Sigma-Aldrich, (Merck, Darmstadt, Germany).

### 4.1. Gel Permeation Chromatography (GPC)

Gel permeation chromatography with multiangle laser light scattering detection (GPC-MALLS) was used to determine the molar mass and molar mass distributions of the XXX polymers. Analysis was performed in THF at 35 °C with a nominal flow rate of 1 mL/min. A column set containing SDV columns from Polymer Standards Service (PSS): guard + 100 Å + 500 Å + 1000 Å + 100,000 Å was used. A differential refractive index detector (Δn-2010 RI WGE Dr. Bures) and a multiangle laser light scattering detector (DAWN HELEOS from Wyatt Technologies) were used in the system. The results were evaluated with ASTRA 5 software (Wyatt Technologies, Santa Barbara, CA, USA).

### 4.2. Nuclear Magnetic Resonance (NMR)

^1^H-NMR spectra were acquired using a Bruker-Advance apparatus (Bruker BioSpin GmbH, Rheinstetten, Germany) operating at 600 MHz with Bruker TOPSPIN 2.0 software using tetramethylsilane (TMS) as an internal standard in deuterated chloroform (CDCl_3_) at 25 °C.

### 4.3. Electrospray Ionization Mass Spectrometry (ESI-MS^n^) Analysis

ESI-MS^n^ analysis was performed using a Thermo LCQ Fleet ion trap mass spectrometer (Thermo Fisher Scientific Inc., San Jose, CA, USA). The samples studied were dissolved in chloroform/methanol (1:1 *v*/*v*), and the solutions were introduced to the ESI source by continuous infusion using the instrument syringe pump at a rate of 10 µL/min. The ESI source of the LCQ was operating at 5.0 kV, and the capillary heater was set to 200 °C. Nitrogen was used as the nebulising gas.

For ESI-MS/MS experiments, monoisotopic ions of interest were isolated in the ion trap and activated using helium damping gas in the mass analyzer to promote collisions induced dissociation (CID). The RF amplitude was set such that the peak height of the molecular ion decreased by at least 50%.

### 4.4. Synthesis of 6-Methyl-ε-caprolactone Monomer

A solution of m-chloroperbenzoic acid (2.47 g, 10 mmol) in methylene chloride (50 cm^3^) was added dropwise to a solution of 2-methylcyclohexanone (1.02 g, 9 mmol) in methylene chloride (30 cm^3^). After 24 h, when the reaction was completed the excess of m-chloroperbenzoic acid was neutralized with saturated aqueous solution of Na_2_SO_3_. The layers were separated, and the organic layer was washed with saturated NaHCO_3_ to pH = 7. Next, the layer was washed with brine and dried over anhydrous MgSO_4_. The crude product was purified by column chromatography with hexane/acetone (4:1) as the eluent. 7-methyl-2-Oxepanone (6-methyl-ε-caprolactone) was obtained with a 93% yield (1.07 g) as a colourless liquid. ^1^H-NMR (600 MHz, CDCl_3_, ppm): 1.19 (3H, (CH_3_)CHO 1.57 (4H, m, CH_2_ (CH_2_CH_2_)CH_2_, 1.89 (2H, m, (CH_2_)CH_2_C(O)O), 2.61 (2H, m, (CH_2_)CHOCH_3_), 4.42 (1H, m, CH_2_(CH)(O)CH_3_). ^13^C-NMR (150 MHz, CDCl_3_, ppm): 22.58, 22.90, 28.27, 35.01, 36.23, 76.83, 175.64.

### 4.5. Homopolymerization of _δ_-Valerolactone

The polymerization of *δ*-valerolactone was conducted as described in our earlier studies [17]. The sample of *δ*-valerolactone (2.0 g, 20 mmol, 50 eq.) was placed in a flame-dried 50 mL two-necked round-bottom flask under nitrogen atmosphere. Ethylene Glycol (0.022 mL, 0.403 mmol, 1.0 eq.) was added, and the flask was placed in a thermostated oil bath at 130 °C. Boric acid (0.025 g, 0.405 mmol, 1.0 eq.) was added with vigorous stirring under a nitrogen atmosphere to start the polymerization. The reaction was quenched after 70 h by the addition of Amberlyst 24 (Sigma-Aldrich, Merck, Darmstadt, Germany) ion exchange resin. The reaction mixture was dissolved in dichloromethane and precipitated with large excess of hexane and subsequently dried under vacuum at room temperature prior to characterization. ^1^H-NMR (600 MHz, CDCl_3_, ppm): 1.68 (4H CH_2_(CH_2_)CH_2_), 2.34 (2H -COCH_2_CH_2_CH_2_CH_2_O-), 3.64 (2H, -COCH_2_(CH_2_)_3_O-), 3.84 (2H, HO(CH_2_)CH_2_O), 4.02 (4H, -COO(CH_2_)_2_CH_2_O), 4.20 (2H, O(CH_2_)CH_2_OH) and 4.29 (4H, O(CH_2_)CH_2_OH).

### 4.6. Copolymerization of _δ_-Valerolactone with 6-Methyl-ε-caprolactone

The copolymerization of *δ*-valerolactone with 6-methyl-ε-caprolactone was conducted as described in earlier studies [17,18]. Briefly, *δ*-valerolactone (1.68 g, 13 mmol, 32 eq.) was placed in a flame-dried 50 mL two-necked round-bottom flask under nitrogen atmosphere. Ethylene Glycol (0.022 mL, 0.403 mmol, 1.0 eq.) was added, and the flask was placed in a thermostated oil bath at 130 °C. Boric acid (0.025 g, 0.405 mmol, 1.0 eq.) was added with vigorous stirring under an argon atmosphere for 24 h to start the polymerization and 6-methyl-ε-caprolactone (0.30 mL, 2.4 mmol, 6 eq.) was added to the reaction mixture. The reaction was allowed to run for another 70 h and was quenched by the addition of Amberlyst 24 (Sigma-Aldrich, Merck, Darmstadt, Germany.) ion exchange resin. The reaction mixture was dissolved in dichloromethane and precipitated twice with large excess of hexane, and subsequently dried under vacuum at room temperature prior to characterization. ^1^H-NMR (600 MHz, CDCl_3_, ppm): 1.20 (3H, (CHOH(CH_3_)), 1.68 (4H CH_2_(CH_2_)CH_2_), 2.34 (2H -COCH_2_CH_2_CH_2_CH_2_O-), 3.64 (2H, -COCH_2_(CH_2_)_3_O-), 3.84 (2H, HO(CH_2_)CH_2_O), 4.02 (4H, -COO(CH_2_)_2_CH_2_O), 4.20 (2H, O(CH_2_)CH_2_OH) and 4.29 (4H, O(CH_2_)CH_2_OH) 5.30 (1H, CH_2_(CH_2_)CH_2_).

## Figures and Tables

**Table 1:** Molar masses and dispersity of the PdVL and P(dVL-co-mCL) telechelic (co)polymers ^a^.

Sample	M_n_,NMR (g/mol)	M_n_,GPC (g/mol)	M_w_/M_n_
1-PdVL	1500	1500	1.07
2-PdVL	4400	5000	1.35
3-PdVL	8900	9700	1.11
1-P(dVL-co-mCL)	1200	1200	1.78
2-P(dVL-co-mCL)	3200	3700	1.83

^a^ Polymerizations were carried out in bulk at 130 °C up to 100% conversion of monomer; M_n_ (NMR) and M_n_ (GPC) were determined via ^1^H NMR spectroscopic analysis in CDCl_3_ and GPC analysis in chloroform (polystyrene as standards), respectively. M_w_/M_n_ is the dispersity determined by GPC.

## Data Availability

The data presented in this study are available on request from the corresponding author. The data are not publicly available due to the potential technological application.
